# Post-Stroke Experiences and Rehabilitation Needs of Community-Dwelling Chinese Stroke Survivors: A Qualitative Study

**DOI:** 10.3390/ijerph192316345

**Published:** 2022-12-06

**Authors:** Janita Pak Chun Chau, Suzanne Hoi Shan Lo, Laveeza Butt, Surui Liang

**Affiliations:** Faculty of Medicine, Nethersole School of Nursing, The Chinese University of Hong Kong, Hong Kong, China

**Keywords:** stroke, post-stroke rehabilitation, exercise, physical activity, quality of life, qualitative

## Abstract

Stroke survivors encounter significant limitations in daily life activities and face increased risk of health complications such as stroke recurrence. Considering the escalating demand for personalised community rehabilitation services, this qualitative study was conducted to understand the current recovery experiences, needs, and expectations of community-dwelling stroke survivors. Fifty stroke survivors were recruited from two tertiary teaching hospitals and community centres in two provinces in mainland China. Semi-structured interviews were carried out, and participants were asked to describe their experiences of stroke, current lifestyles, exercise habits, and rehabilitation needs and expectations. Resulting data were thematically analysed. The majority of participants were first-time stroke survivors (80%) and lived with their family or caregivers (92%). Four main themes and twelve sub-themes emerged from the data: (1) shifts in social life, (2) shaken sense of self and perceived helplessness, (3) complex rehabilitation needs, and (4) perceptions and patterns of physical activity. Findings suggest that though survivors recognised their need for further rehabilitation, their demands remained unmet due to a combination of personal and external factors such as limited mobility and the absence of supportive companions and accessible facilities. The enhancement and diversification of home rehabilitation strategies are therefore necessary to make community rehabilitation more accessible and equitable.

## 1. Introduction

Stroke is a leading cause of death and disability around the world, resulting in over 143 million disability-adjusted life years (DALYS) lost per year [1]. The disease causes significant disruptions in survivors’ lives as they experience a range of physical, psychological, and cognitive impairments. In the long term, survivors may also suffer from poor quality of life due to reduced physical activity, low social participation, and the development of cardiovascular diseases [2,3], all of which may contribute to negative health outcomes such as stroke recurrence and mood disorders [4,5,6]. It is therefore necessary for discharged stroke survivors to have access to effective rehabilitation and community care services in order to support their physical and psychological recovery and promote positive health outcomes.

Evidence shows that maintaining a healthy lifestyle through appropriate rehabilitation regimens such as regular physical exercise can improve long-term health by reducing the impact of post-stroke sequelae and diseases [7,8,9]. However, inpatient rehabilitation in the post-acute stage is often insufficient in fully addressing continuing health needs, such as survivors’ recovery of functional independence, quality of life, and social participation [10,11]. Moreover, the transition from hospital to community settings has also been poorly evaluated by survivors who reported a sense of abandonment and dissatisfaction with existing inpatient and community services as they perceived a lack of professional attention to their personal recovery needs and goals [12,13]. As such, more individualised and targeted care services are required to cater to the lasting physical and psychological health demands of community-dwelling stroke survivors.

Patient involvement in rehabilitation planning can potentially lead to more favourable recovery outcomes [14]. In China, less than 12% of patients undergo rehabilitation within 1 week post-stroke, and nearly 43% do not undergo any rehabilitation at all, demonstrating both a lack of awareness regarding the benefits of early rehabilitation and possible barriers to accessing rehabilitation services [15]. Moreover, as standardised stroke care is still under development, healthcare providers’ stroke knowledge still remains inadequate, and there is insufficient integration and coordination of stroke care teams and systems, thus resulting in a lack of comprehensive care for stroke survivors, who ultimately have to rely on themselves or family caregivers for rehabilitation [16]. As such, recognising that survivors may have unmet needs, this qualitative study was carried out to understand the current recovery experiences, needs, and expectations of Chinese community-dwelling stroke survivors. Though several qualitative studies have explored the experiences and demands of stroke caregivers in China, there is a paucity of recent studies focusing on the experiences of survivors themselves. In order to meet the wide variety of unmet survivor demands, which include physical, psychosocial, information, and other supplementary support needs, survivors’ lived experiences were thus investigated to inform the design of more relevant and effective rehabilitation services.

## 2. Materials and Methods

### 2.1. Design

A descriptive qualitative study with semi-structured interviews was carried out. This study design was chosen as it is grounded in the principles of naturalistic inquiry [17], thus allowing researchers to develop an understanding of the subjective realities of participants. As such, direct insights can be elicited from key stakeholders to support the enhancement of health policy and services [18].

### 2.2. Participants

Participants were recruited through purposive sampling from two tertiary teaching hospitals and community centres in two provinces in mainland China. A PhD in nursing student who is also a registered nurse screened stroke patients and individually invited potential participants to join the study. Participants were included if they were: (1) aged 18 or above, (2) diagnosed with a first-ever/recurrent ischaemic or haemorrhagic stroke, (3) able to communicate in Chinese, and (4) residing in the community. Survivors that lost the ability to communicate due to aphasia were excluded.

### 2.3. Data Collection and Analysis

Fifty stroke survivors agreed to join the study. Semi-structured interviews were carried out according to an interview guide developed by the research team, which has comprehensive experience in stroke rehabilitation and gerontology care. Pilot interviews were conducted with three participants, and the interview guide was refined accordingly (Table 1). Interviews were conducted by the PhD student who also has substantial experience in qualitative research and stroke rehabilitation. A total of 41 interviews took place face-to-face in Chinese in a private room at the hospital, 3 took place in participants’ homes, and 6 interviews were conducted virtually via online calls. The interviews lasted for 15–30 min and were audio-recorded with participants’ consent. Data collection concluded once data saturation was achieved. Interviews were transcribed verbatim by the PhD student, and the resulting data were analysed in accordance with Braun and Clarke’s six-step framework for thematic analysis [19]. Transcripts were first read and re-read to increase familiarisation with the data. The data were then coded line-by-line inductively by a research assistant with experience in qualitative research, and the resulting codes were reviewed and modified along the process. Finalised codes were reviewed by all research team members and then grouped into themes and sub-themes based on the salience of the data. The resulting findings were reviewed and confirmed by all authors.

### 2.4. Trustworthiness of Data

Lincoln and Guba’s (1985) four principles of trustworthiness were applied to ensure the reliability of the data [20]. Credibility was established through persistent observation and regular meetings between research team members to discuss data interpretation and achieve consensus regarding emerging findings. Thick descriptions of participants’ post-stroke rehabilitation experiences, including contextual factors and direct quotations from participants, were provided to ensure transferability. An audit trail of the coding and categorisation process was maintained to establish dependability and confirmability of the data.

### 2.5. Ethical Consideration

This study was approved by the Survey and Behavioural Research Ethics Committee of The Chinese University of Hong Kong (Ref. No.: SBRE-18-469). Participants’ rights and safety were safeguarded according to the Declaration of Helsinki. During recruitment, the study purpose and procedures were explained to participants and written informed consent was obtained. Participants were informed of their right to refuse to participate or withdraw from the study at any time. All data were anonymised and stored securely in encrypted files. Access to the data was only available to research team members.

## 3. Results

Fifty stroke survivors were interviewed for this study. The mean age of the participants was 61.58 years (SD: 13.2), and the majority (68%) were men. Most participants (80%) had an education level of secondary or less and were employed pre-stroke (78%), whereas all participants were either unemployed or retired after stroke. The majority (92%) of participants lived with their family or caregivers and did not have an elevator in their residence. Hypertension was the most common co-morbidity with a prevalence of 48%, followed by diabetes at 14% and heart diseases at 6%. Over half of the participants (54%) did not use assistive aids. Ischaemic stroke was more common (60%) than haemorrhagic stroke (40%), and most participants (80%) had experienced stroke once only. Half of the participants (54%) reported disability in the left limbs, and the other half (42%) in their right limbs. Meanwhile, two participants experienced disability on both sides of their bodies. Further details of participant characteristics can be found in Table 2.

Four themes and twelve sub-themes emerged from the data and are summarised in Table 3. A summary table of themes and representative participant quotes is available as a Supplementary Materials (Table S1).

Theme 1. Shifts in Social Life
Sub-Theme 1.1 Reversal of Family Role

The survivors expressed a strong sense of having an altered social identity as the nature of their relationships began to change due to the sudden setbacks in their physical and cognitive abilities. Particularly in terms of their family roles, survivors felt that their responsibilities had become reversed, going from being providers or caregivers who could operate independently to becoming care recipients reliant on others.

*“It was a major shock when I couldn’t speak clearly. On top of that, I couldn’t move and needed my family to take care of me…it was a big blow.”*

(P10, 56 years, male)

*“I used to earn and buy things for my wife and kids, but now I don’t have a source of income so I can’t do that anymore.”*

(P49, 50 years, male)

Besides their personal perception of reversed family roles, participants also revealed how some family relationships had become strained due to feelings of resentment and frustration arising from new responsibilities.

*“My husband says he feels tied down by me since he can’t go out to do what he wants.”*

(P2, 49 years, female)

Sub-Theme 1.2 Reduced Community and Social Contact

Besides facing changes in family dynamics, participants also experienced a heightened sense of isolation from their community and friends resulting from physical and speech impairments, which discouraged them from going outdoors and conversing with others as they normally would.

*“I used to go out and do activities with other people but now I just sit alone… I can’t walk so I have to stay [at home] by myself.”*

(P1, 75 years, female)

*“There’s a vegetable market downstairs and I liked to go down and buy things… These were my hobbies but now I can’t do them anymore, I’ve started to feel lonely…”*

(P21, 77 years, female)

*“I feel very bored by myself at home. I wish people would come and hang out with me.”*

(P2, 49 years, female)

Feelings of shame or embarrassment about their disability were also prevalent, particularly among those with speech impairments. Since participants were afraid of sounding different and being judged, this became another factor that caused them to avoid social interaction.

*“I don’t want to socialise with anyone anymore because I can’t speak [properly] now. I used to be very talkative but now I don’t want to speak to anyone.”*

(P46, 65 years, male)

*“I feel like I sound a bit strange so I prefer not talk to others.”*

(P1, 75 years, female)

Theme 2. Shaken Sense of Self and Perceived Helplessness

When reflecting upon their disabilities and radically altered lives post-stroke, participants frequently felt upset and revealed a sense of inadequacy, reflecting a deterioration in self-image and dissatisfaction with their circumstances. Specifically, participants expressed dissatisfaction regarding their reduced ability to support their families, not just financially but in terms of depriving them of a healthy family member.

*“I felt really sad because I still have two little children… I felt very sorry towards them that I’ve got this illness and whenever I saw them I’d cry…I couldn’t accept the situation… me being jobless and not earning and my wife taking a year off to take care of me. When I looked at this situation, it’s really upsetting.”*

(P50, 41 years, male)

*“There weren’t many patients [in the ward] and it felt like everyone else was alive and kicking…when I was discharged, I cried because I felt alone and wondered why it had to happen to me.”*

(P3, 57, female)

Participants also felt incapacitated due to activity restrictions caused by stroke, especially when they contrasted their current situation with their comparatively active pre-stroke lifestyles.

*“[Before stroke] I used to wake up at 5 a.m. to go hiking. On the way home, I would buy a bun and return after eating. But now, I don’t go anymore and will only go out to walk if I’m urged.”*

(P30, 63 years, male)

*“It felt like being locked up in prison… can’t go out, can’t do anything [you want].”*

(P7, 52 years, male)

Some participants felt overwhelmed at the prospect of living with their disabilities, reflected by their revelation of major depressive symptoms.

*“I was suicidal… almost jumped off the building. It felt like my life was over and I wondered, why does it have to be like this? How am I going to live my life with stroke?”*

(P49, 50 years, male)

Theme 3. Complex Rehabilitation Needs
Sub-Theme 3.1 Physical and Psychosocial Recovery

As most participants were in the process of rehabilitation, they expressed a strong desire to recover functional capabilities that would allow them to resume self-management, such as being able to prepare their own meals, reduce dependence on others, and restart former activities.

*“I want to recover quickly and take care of my family. I want to recover by myself and at least be able to move my hands… make some dumplings.”*

(P15, 72 years, female)

*“I’m just waiting for the day when I can prepare my meals by myself without needing help from others.”*

(P1, 75 years, female)

They also emphasised a need for counselling services to deal with the psychological impacts of stroke and suggested that door-to-door volunteer services could be arranged to provide companions to less mobile survivors in order to increase their social interactions.

*“We need more help in terms of our physical and emotional health… I still have some anxiety and whenever an issue arises, I will become anxious because I can’t act immediately like I used to before.”*

(P12, 45 years, male)

*“Door-to-door rehab services would be beneficial as it would be good to have someone to accompany us… I feel bored at home by myself.”*

(P48, 51 years, male)

Strong family and peer support were also noted as a major rehabilitation need, particularly for older adults who were more vulnerable to social isolation and physical injuries due to participation restrictions and poorer mobility. Moreover, participants felt that communication with peers would also be productive as they could exchange first-hand stroke and rehabilitation knowledge and seek each other out for motivation and psychological support.

*“We need family affection… Older people need to get encouragement and warmth from their families.”*

(P21, 77 years, female)

*“Hope to have someone to talk to and share our experiences… if [other survivors] are recovering well then I could learn from them and improve my recovery. If I just stay at home, I definitely won’t recover well and I won’t have a lot of information.”*

(P25, 76 years, male)

*“After getting to understand this disease, I started to meet more peers on Douyin, WeChat and other survivors in the community. I’d talk to them about stroke-related things…”*

(P50, 41 years, male)

Sub-Theme 3.2 Home Guidance from Professionals and Volunteers

Regarding rehabilitation post-discharge, participants hoped that they could receive direct guidance from rehabilitation and healthcare workers at their homes. Without the relevant guidance and direction, participants felt lost and unsure about the kind of rehabilitation regimen that would be conducive to their recovery.

*“We want [healthcare] volunteers to teach us about rehabilitation since we don’t understand it very well. They’re professional and could teach us how to recover.”*

(P41, 68 years, female)

*“Guidance and advice provided by rehabilitation experts is important. We will pay attention to them…after hospital discharge, it’d be good if professional rehabilitation workers could help us do home rehabilitation instead of us trying to do it by ourselves. It would save us a lot of time and effort.”*

(P11, 55 years, male)

*“I hope that more doctors can visit [the elders] in our community regularly… There should be more on-site services.”*

(P38, 59 years, female)

Sub-Theme 3.3 Physically Accessible Rehabilitation Spaces

Since several participants had to travel to hospitals or clinics outside their community/neighbourhoods to receive rehabilitation, they expressed a sense of fatigue with the amount of commuting involved and expressed a need for more rehabilitation facilities closer to their homes or for rehabilitation strategies that were home-based.

*“It just feels like I’m always going back and forth between the hospital and home.”*

(P17, 55 years, female)

Besides issues with the location of rehabilitation facilities, survivors were enthusiastic about the prospect of socialising and exercising together. They felt that it would be helpful if dedicated spaces such as activity rooms could be arranged to simultaneously act as public fitness centres as well as locations for socialisation and mutual learning among peer survivors.

*“I want to see how [other survivors’] rehabilitation exercises are working for them. If they are exercising well and it’s benefitting them, I would want to learn from them.”*

(P2, 49 years, female)

Sub-theme 3.4 Institutional Support

Participants reflected positively on the support provided by community organisations in promoting mobility and survivor reintegration. Though some participants were unaware of the services available, one noted that the Disabled Persons’ Federation were proactive in contacting people who may potentially need their services and in offering relevant support. For example, several participants were aware that the Federation could make home modifications to increase environmental safety, such as installing handrails in bathrooms.

*“Last time we went to the Disabled Persons’ Federation, they were quite good as they asked us if we wanted to install any handrails in the shower… They also asked if we needed any assistive devices such as wheelchairs or anything else.”*

(P35, 44 years, male)

*“The door-to-door volunteer service provided by Disabled Persons’ Federation is quite good. I felt supported by their measures and I feel like their support can reduce pressure on me.”*

(P24, 85 years, female)

However, not all participants were aware of such community services and at times found that they were insufficient in addressing their daily needs. For instance, there is evidence of community care worker shortages as some participants were unable to find caregivers when they needed them urgently during the acute stage post-stroke. Especially as participants want to avoid placing caregiving responsibilities on their families, there is a high demand for community care workers who could temporarily provide daily life support while they recover gradually.

*“My family members and I tried to look for [a caregiver] in the start but we couldn’t find anyone…”*

(P28, 55 years, female)

*“Since both my kids need to work and earn money, I needed someone to accompany me [after I was discharged].”*

(P25, 76 years, male)

Theme 4. Perceptions and Patterns of Physical Activity
Sub-Theme 4.1 Perceived Benefits of Physical Activity

In general, survivors recognised the value of regular physical exercise and noted that it had helped them improve their mobility.

*“Cycling helps. I think it has helped my arms and legs to recover gradually.”*

(P8, 44 years, female)

*“[After exercise] now I can lift this hand up. In the beginning, I couldn’t lift it no matter how hard I tried.”*

(P1, 75 years, female)

*“When I was at the hospital, I couldn’t walk a single step but after doing two or three different types of rehab exercises, I could finally start to walk.”*

(P2, 49 years, female)

However, although the value of exercise was recognised, some older participants had a more passive stance regarding their recovery and did not feel the need to make additional rehabilitation efforts.

*“I think that at this old age, it is already enough if I can eat well, live well, and not starve.”*

(P1, 75 years, female)

Sub-Theme 4.2 Walking and Home Exercise as the Main Form of Physical Activity

In terms of the types of rehabilitation exercise, walking was the most common form of physical activity reported by participants. However, depending on the participants’ physical condition, some were able to walk longer and further, while others were limited to short walks. Some participants had also purchased home exercise equipment such as pulleys, slant boards, and horizontal bars that could be attached to doors.

*“I’ll wake up at 6:30 in the morning and go out for a walk and some exercise before breakfast. I’ll exercise in the public square.”*

(P17, 55 years, female)

*“I just do some little exercise at home… If I go out, I’ll just walk a bit in a straight line, stretch my neck and twist my hands.”*

(P20, 73 years, female)

Sub-Theme 4.3 Barriers to Use of Public Exercise Equipment

While some participants were able to reach and use public exercise facilities in parks or health centres, a mismatch in the needs of stroke survivors and public facilities was also revealed as others were unable to use the community equipment due to issues of accessibility or because the equipment was not appropriate for their type of disability.

*“I don’t use the community equipment because I feel like they aren’t very suitable for my body type. They don’t seem very safe.”*

(P20, 73 years, female)

Other participants pointed out features in their built environment, such as the lack of elevators in their residence, as a barrier to accessing and making use of public exercise facilities.

*“When I was healthy, I would always go downstairs to stretch, do waist twists, exercise… but now I can’t walk anymore and we live on the 7th floor with no elevator…”*

(P35, 44 years, male)

Sub-theme 4.4 Physical Activity Participation Affected by Self-Efficacy

Besides the lack of exercise equipment, participants also deliberately avoided exercise due to cautious attitudes regarding physical activity. Since survivors’ mobility was affected by physical limitations such as limb weakness, they were afraid of injuring themselves or causing inconvenience to others due to falls or other adverse incidents, reflecting low self-efficacy in performing physical activity.

*“I don’t leave home… I’m scared even to go to the backyard… I’m afraid if I squat down and my leg goes numb, what will I do if I can’t get back up?”*

(P15, 72 years, female)

*“I’m quite scared, especially when [my caregiver] is not with me since I’m afraid of injuring myself.”*

(P44, 65 years, female)

On the other hand, participants felt more secure and confident in participating in exercise when accompanied by a caregiver or peer groups, suggesting that companions were a motivating factor in encouraging exercise and could alleviate survivor concerns.

*“If my caregiver takes me for longer walks, then I can walk more. I’ve always felt that this is the most important thing because from the beginning to the present, whenever I couldn’t walk, they insisted on helping me.”*

(P4, 52 years, male)

For example, one participant detailed his experience of going hiking with a group of stroke survivors and expressed his delight at the liberating experience of going on a trip.

*“I went to Chongqing and Guang’an a while ago, met my friends there and went hiking together. We didn’t go on steep roads and mainly stayed on flat paths. It was quite amazing. It’s the first time I’ve gone so far since I got sick.”*

(P50, 41 years, male)

Besides being able to visit the outdoors and have social interaction, this anecdote also demonstrates survivors’ ability to take their collective physical limitations into consideration when planning group activities, suggesting that peer support is desirable in the organisation of effective and appropriate post-stroke community activities.

## 4. Discussion

This study highlights the post-discharge experiences of community-dwelling Chinese stroke survivors, and their persisting care and support needs, and expectations. In accordance with previous studies, survivors expressed challenges in completing daily tasks and revealed insufficient social interaction, both of which negatively affected their psychological health and overall quality of life [21,22]. Additionally, our findings demonstrate a need for more equitable access to care services as home-based rehabilitation strategies emerged as a popular solution to address the limitations of traditional outpatient or community care.

Survivors had a wide range of disabilities and corresponding recovery and care needs, indicating that post-discharge community services should take an individualised approach to adequately address survivor demands. For instance, though efforts such as the installation of public fitness equipment in several residential communities have increased accessibility and facilitated physical exercise, survivors with severely limited physical function still cannot benefit as other environmental barriers, such as the lack of elevators in their buildings, still prevented participation. It is thus necessary to evaluate each survivor’s unique circumstances prior to discharge in order to develop pragmatic plans to optimise their rehabilitation and recovery in the community. In this regard, home-based rehabilitation strategies tailored to survivors’ individual situations can be effective in addressing specific personal needs, with evidence suggesting that home-based interventions, which are developed following an evaluation of survivor goals are equally or more effective than conventional outpatient rehabilitation [23,24].

Besides environmental limitations, survivors’ engagement in physical exercise, as well as social participation, was influenced by psychological factors such as poor self-image and low self-efficacy. As a central concept applied in health sciences, self-efficacy refers to an individual’s belief or confidence in their own ability to achieve a specific task. As stroke often causes life-altering disabilities, survivors face significant psychological distress while adjusting to their “new normal” experience of daily life. In terms of post-stroke exercise and participation specifically, our study reflects previous findings and confirms that fear and anxiety are prevalent as survivors worry about adverse incidents such as falls and demonstrate low confidence in their participation capabilities [25,26]. As such, even if other environmental factors are favourable and conducive to exercise and participation, if survivors’ psychological health is not sufficiently addressed, they may be unable to take advantage of available rehabilitation services and risk poorer health outcomes. This is particularly disadvantageous as though survivors in the current study demonstrated high awareness of the necessity of physical activity for their recovery, if they were unable to overcome psychological barriers, they may be deterred from following through with the demands of rehabilitation. Community counselling services should therefore be strengthened to help survivors manage their emotional health, improve their confidence, and encourage participation in rehabilitation, physical exercise, and social activities. Specifically, home visits by volunteers could be arranged to provide company to survivors, as suggested by survivors in the current study. Especially as previous evidence supports the advantages of volunteer visits in reducing mood disorders and increasing community reintegration [27,28], more efforts could be dedicated to developing volunteer networks and equipping them with relevant skills to cater to survivors’ psychological and rehabilitation needs.

Aside from volunteer involvement, survivors’ experiences also reveal a need for stronger social support networks to facilitate them in their recovery and re-engagement in daily life activities. Noting the importance of physical activity in rehabilitation for example, survivors felt more confident in their mobility when they had a companion who they could rely on in case of potential mishaps, echoing findings from past studies, which note that support from partners or family members was a key facilitator of physical activity [29,30]. Moreover, evidence also suggests that rehabilitation interventions that actively involve survivor family caregivers can improve key outcomes such as functional independence and psychological health [31,32]. Furthermore, considering the shortage of healthcare workers, peers could also be recruited to provide rehabilitation guidance for community survivors, with evidence confirming the benefits of peer coaching sessions and support groups on both physical and psychological health [33,34]. As such, post-discharge rehabilitation strategies should also seek to involve peers and empower people closest to the survivors, such as family members and caregivers, so as to create a favourable environment for survivor rehabilitation.

Additionally, our findings also shed light on the needs of survivors with speech disabilities, which are often hidden and associated with worse long-term health outcomes [35]. Though survivors mentioned that they would practice speaking with their caregivers, no formal speech rehabilitation strategies were reported, suggesting an inadequacy of speech therapy services also noted in other areas around the world [36,37]. Moreover, the inability to speak “properly” not only harmed survivors’ self-image but also reduced their desire for social interaction. Considering the central function of speech in driving social participation and functional independence, there is an urgent demand for speech therapy services in the community in order to improve survivors’ capability of expression and, more importantly, raise their confidence in communication regardless of their speaking skills. At the same time, educational courses on effective communication with people with speech disabilities may be organised for interested stakeholders such as caregivers, family, and friends in order to reduce the burden of re-learning on stroke survivors and encourage positive interpersonal communication within their social circles.

Our findings reflect previous evidence on community stroke rehabilitation and indicate that a diverse range of stroke services are required to meet current rehabilitation and daily care demands [38]. In particular, environmental and social restrictions caused by disabilities need to be addressed in order to facilitate survivors’ rehabilitation. Moreover, though survivors have found ways to adapt and persist in their rehabilitation through self-management strategies, current services can be enhanced to provide further support and facilitate post-stroke community living.

## 5. Limitations

Participant recruitment was limited to a relatively small geographical area and to stroke survivors with mild to moderate disabilities only, which may reduce the generalisability of the findings. Furthermore, as participants with severe speech disabilities, such as global aphasia, were excluded from the study, the needs of this specific group of survivors may be underexplored. In addition, as there may be sociocultural influences that affect the length and complexity of participant responses [39], the relatively short duration of some interviews may limit the depth of the findings. Future studies could address this by employing measures to improve rapport, trust, and level of comfort between interviewers and participants in order to elicit more comprehensive responses [39]. Additionally, though the physical environment exerts a significant influence on stroke survivors’ needs, limited data were collected regarding the built environment, which could have provided more specific insights into how survivors’ experiences were dependent on their environment. As such, future studies may focus on investigating environmental factors and identifying their associations with survivors’ unmet needs. Moreover, as survivors’ needs for different types of rehabilitation and support vary depending on the time since stroke, this is another factor that can be considered in future studies in order to better match survivors with the specific types of services required.

## 6. Conclusions

Our study gives insights into the current rehabilitation experiences and needs of Chinese community-dwelling stroke survivors, with a focus on factors related to engagement with physical exercise and participation. While survivors revealed that they had a variety of post-discharge care needs, home-based rehabilitation emerged as the most popular recommendation to address the wide range of survivor needs. These include home exercise guidance from rehabilitation professionals or volunteers, door-to-door services providing basic household support, counselling services, and home modifications. Survivors’ short- and long-term needs should be assessed pre-discharge in order to make adequate preparations for community reintegration and to match survivors with the required rehabilitation and auxiliary support services. Future studies may investigate survivors’ experiences with specific types of community rehabilitation services in order to identify service areas requiring improvement.

## Figures and Tables

**Table 1:** Questions in semi-structured interview guide.

Please share your recovery experience after discharge.
2. Please share your experience of managing your rehabilitation regimen at home.
3. Please share your experience in managing your emotions.
4. Please share how you manage your role in your family and society.
5. What are/were your priority health needs in the initial period after discharge?
6. What are/were your priority health needs during the first three years after discharge?
7. Please share your experience of using existing health services in the community.
8. Based on your experience, which health services do you think are most helpful to you?
9. Based on your experience, what types of health services do you think are not adequate at present?
10. What are the factors that affect your use of health services?
11. Based on your experience, what types of services or support do you require to better meet your health needs?
12. Do you have anything we haven’t mentioned that you would like to share?

**Table 2:** Characteristics of the study sample (N = 50).

	Mean (SD)/n (%)
**Sociodemographic characteristics**	
Age (years) †	61.58 (13.2)
Sex	
Male	34 (68%)
Female	16 (32%)
Educational level	
No formal education/primary	21 (42%)
Secondary	20 (40%)
Post-secondary or above	9 (18%)
Marital status	
Married	49 (98%)
Divorced/widowed	1 (2%)
Employment pre-stroke	
Employed	39 (78%)
Unemployed	7 (14%)
Retired	4 (8%)
Employment post-stroke	
Employed	0 (0%)
Unemployed	50 (100%)
Living Situation	
Living alone	4 (8%)
Living with caregiver	2 (4%)
Living with family	44 (88%)
Financial Assistance	
No	39 (78%)
Yes	11 (22%)
Elevator in residence	
No	46 (92%)
Yes	4 (8%)
**Clinical characteristics**	
Average time since most recent stroke (years) †	5.08 (5.90)
History of hypertension	
No	26 (52%)
Yes	24 (48%)
History of diabetes	
No	43 (86%)
Yes	7 (14%)
History of heart disease	
No	47 (94%)
Yes	3 (6%)
Assistive aids used	
None	27 (54%)
Walking cane and/or Wheelchair	23 (46%)
Type of stroke	
Ischaemic stroke	30 (60%)
Haemorrhagic stroke	20 (40%)
Affected side	
Left	27 (54%)
Right	21 (42%)
Both	2 (4%)
Affected limbs	
Upper	8 (16%)
Lower	1 (2%)
Both	41 (82%)
Stroke frequency	
One	40 (80%)
Two or more	10 (20%)

Data marked with † are presented as mean (standard deviation). All others are presented as frequency (%).

**Table 3:** Summary of themes emerging from the data.

Main Themes	Sub-Themes
Shifts in social life	1.1 Reversal of family role
	1.2 Reduced community and social contact
2. Shaken sense of self and perceived helplessness	/
3. Complex rehabilitation needs	3.1 Physical and psychosocial recovery
	3.2 Home guidance from professionals and volunteers
	3.3 Physically accessible rehabilitation spaces and solutions
	3.4 Institutional support
4. Perceptions and patterns of physical activity	4.1 Perceived benefits of physical activity
	4.2 Walking and home exercise as the main form of physical activity
	4.3 Barriers to use of public exercise equipment
	4.4 Physical activity participation affected by self-efficacy

## Data Availability

The data that support the findings of this study are available from the corresponding author upon reasonable request.

## Supplementary Materials

The following supporting information can be downloaded at: https://www.mdpi.com/article/10.3390/ijerph192316345/s1. Table S1: Summary of themes and representative participant quotes.
