# Supplementary material for: Post-Stroke Experiences and Rehabilitation Needs of Community-Dwelling Chinese Stroke Survivors: A Qualitative Study
Source: Int J Environ Res Public Health. 2022 Dec 6;19(23):16345. doi: 10.3390/ijerph192316345 (PMC9741278; doi:10.3390/ijerph192316345)
Supplement: Supplementary file 1 [file ijerph-19-16345-s001.zip › ijerph-1996777-supplementary.pdf]

Table S1: Summary of themes and representative participant quotes

Main themes	Sub-themes	Participant quotes
1. Shifts in social life	1.1 Reversal of family role	*"It was a major shock when I couldn't speak clearly. On top of that, I couldn't move and needed my family to take care of me…it was a big blow."* (P10, 56 years, male)<br><br>*"I used to earn and buy things for my wife and kids, but now I don't have a source of income so I can't do that anymore."* (P49, 50 years, male)
	1.2 Reduced community and social contact	*"I used to go out and do activities with other people but now I just sit alone… I can't walk so I have to stay [at home] by myself."* (P1, 75 years, female)<br><br>*"There's a vegetable market downstairs and I liked to go down and buy things… These were my hobbies but now I can't do them anymore, I've started to feel lonely…"* (P21, 77 years, female)
2. Shaken sense of self and perceived helplessness	/	*"I felt really sad because I still have two little children… I felt very sorry towards them that I've got this illness and whenever I saw them I'd cry…I couldn't accept the situation… me being jobless and not earning and my wife taking a year off to take care of me. When I looked at this situation, it's really upsetting."* (P50, 41 years, male)<br><br>*"[Before stroke] I used to wake up at 5 a.m. to go hiking. On the way home, I would buy a bun and return after eating. But now, I don't go anymore and will only go out to walk if I'm urged."* (P30, 63 years, male)
3. Complex rehabilitation needs	3.1 Physical and psychosocial recovery	*"I want to recover quickly and take care of my family. I want to recover by myself and at least be able to move my hands… make some dumplings."* (P15, 72 years, female)<br><br>*"We need more help in terms of our physical and emotional health… I still have some anxiety and whenever an issue arises, I will become anxious*

		because I can't act immediately like I used to before." (P12, 45 years, male)<br><br>*"Hope to have someone to talk to and share our experiences… if [other survivors] are recovering well then I could learn from them and improve my recovery. If I just stay at home, I definitely won't recover well and I won't have a lot of information."* (P25, 76 years, male)
	3.2 Home guidance from professionals and volunteers	*"Guidance and advice provided by rehabilitation experts is important. We will pay attention to them…after hospital discharge, it'd be good if professional rehabilitation workers could help us do home rehabilitation instead of us trying to do it by ourselves. It would save us a lot of time and effort."* (P11, 55 years, male)<br><br>*"I hope that more doctors can visit [the elders] in our community regularly… There should be more on-site services."* (P38, 59 years, female)
	3.3 Physically accessible rehabilitation spaces and solutions	*"It just feels like I'm always going back and forth between the hospital and home."* (P17, 55 years, female)<br><br>*"I want to see how [other survivors'] rehabilitation exercises are working for them. If they are exercising well and it's benefitting them, I would want to learn from them."* (P2, 49 years, female)
	3.4 Institutional support	*"Last time we went to the Disabled Persons' Federation, they were quite good as they asked us if we wanted to install any handrails in the shower… They also asked if we needed any assistive devices such as wheelchairs or anything else."* (P35, 44 years, male)<br><br>*"The door-to-door volunteer service provided by Disabled Persons' Federation is quite good. I felt supported by their measures and I feel like their support can reduce pressure on me."* (P24, 85 years, female)

4. Perceptions and patterns of physical activity	4.1 Perceived benefits of physical activity	*"[After exercise] now I can lift this hand up. In the beginning, I couldn't lift it no matter how hard I tried."* (P1, 75 years, female)<br><br>*"When I was at the hospital, I couldn't walk a single step but after doing two or three different types of rehab exercises, I could finally start to walk."* (P2, 49 years, female)
	4.2 Walking and home exercise as the main form of physical activity	*"I'll wake up at 6:30 in the morning and go out for a walk and some exercise before breakfast. I'll exercise in the public square."* (P17, 55 years, female)<br><br>*"I just do some little exercise at home… If I go out, I'll just walk a bit in a straight line, stretch my neck and twist my hands."* (P20, 73 years, female)
	4.3 Barriers to use of public exercise equipment	*"I don't use the community equipment because I feel like they aren't very suitable for my body type. They don't seem very safe."* (P20, 73 years, female)<br><br>*"When I was healthy, I would always go downstairs to stretch, do waist twists, exercise… but now I can't walk anymore and we live on the 7th floor with no elevator…"* (P35, 44 years, male)
	4.4 Physical activity participation affected by self-efficacy	*"I don't leave home… I'm scared even to go to the backyard... I'm afraid if I squat down and my leg goes numb, what will I do if I can't get back up?"* (P15, 72 years, female)<br><br>*"If my caregiver takes me for longer walks, then I can walk more. I've always felt that this is the most important thing because from the beginning to the present, whenever I couldn't walk, they insisted on helping me."* (P4, 52 years, male)
